# Effects of Mini-Basketball Training Program on Social Communication Impairment and Executive Control Network in Preschool Children with Autism Spectrum Disorder

**DOI:** 10.3390/ijerph18105132

**Published:** 2021-05-12

**Authors:** Sixin Yang, Zhimei Liu, Xuan Xiong, Kelong Cai, Lina Zhu, Xiaoxiao Dong, Jingui Wang, Hao Zhu, Yifan Shi, Aiguo Chen

**Affiliations:** 1 College of Physical Education, Yangzhou University, Yangzhou 225127, China; MX120200467@yzu.edu.cn (S.Y.); MX120170354@yzu.edu.cn (Z.L.); movmu7@sina.com (X.X.); MX120170353@yzu.edu.cn (K.C.); DX120190065@yzu.edu.cn (X.D.); MX120170362@yzu.edu.cn (J.W.); MX120180365@yzu.edu.cn (H.Z.); MX120190380@yzu.edu.cn (Y.S.); 2 Institute of Sports, Exercise and Brain, Yangzhou University, Yangzhou 225127, China; 3 School of Physical Education and Sports Science, Beijing Normal University, Beijing 100000, China; zhulina827@mail.bnu.edu.cn; 4 Chinese-Polish Laboratory of Sport and Brain Science, Yangzhou University, Yangzhou 225127, China

**Keywords:** mini-basketball, autism spectrum disorder, social communication, executive control network, functional connectivity

## Abstract

This study evaluated the effect of a 12-week mini-basketball training program (MBTP) on social communication (SC) and the executive control network (ECN) in preschool children with autism spectrum disorders (ASD). We finally assigned 30 preschool children with ASD to an experiment group (*n* = 15, 12 males, 3 females) or a control group (*n* = 15, 13 males, 2 females). The experiment group participated in a 12-week MBTP (40-min sessions per day, 5 days a week), while the control group only received the institutional routine behavioral rehabilitation intervention. The SC of preschool children with ASD was measured using the Social Responsiveness Scale, Second Edition (SRS-2), whereas functional connectivity (FC) of the ECN was assessed using resting-state functional magnetic resonance imaging (rs-fMRI) at pre-and post-test. Our results showed that SC exhibited significant improvement in the intervention group, especially in SRS-2 total score, social cognition, and social communication. We found significantly enhanced functional connectivity between the right cerebellum and left inferior frontal gyrus in the experimental group, while functional connectivity between the left middle temporal gyrus and right cerebellum were decreased in the control group. Furthermore, there were no significant correlations between the change in SC scores and FC of the ECN. Altogether, this study provides valuable insights that a 12-week MBTP improves SC and functional connectivity of the ECN in preschool children with ASD. We further inferred that neural mechanisms might be associated with changing the ECN of preschool ASD children caused by the 12-week MBTP.

## 1. Introduction

Autism spectrum disorder (ASD) is a neurodevelopmental disorder of unknown etiology [1]. According to the latest statistics from the U.S. Centers for Disease Control and Prevention (CDC), the prevalence of ASD has increased to 1 in 54 children [2]. The rapidly growing incidence of this disease has aroused public concern. Social communication (SC) impairment is one of the main characteristics of ASD, and manifests as deficits in social cognition, pragmatics, language processing, and verbal and nonverbal communication [3]. Overwhelming evidence shows that SC deficit limits the individual development of ASD children, seriously affecting the quality of their family life [4], as well as posing a substantial economic burden on society [5]. Recent research suggests that early interventions in children diagnosed with ASD before age 3 can significantly improve cognitive, social, and adaptive functioning in children with ASD [6]; therefore, early intervention and treatment in preschool is desperately needed.

Recently, physical exercise programs have been widely used in the therapy of children with ASD, and more importantly, physical exercise has a significant effect on improving SC impairment [7,8,9,10,11,12,13]. Among them, mini-basketball has the inherent characteristics of basketball and is designed for children under 12 years old, which is parallel with the physical and mental developmental characteristics of children. Mounting evidence has demonstrated that a mini-basketball training program (MBTP) can effectively improve the core symptoms of children with ASD, as well as promote the development of physical fitness [14], cognitive function [15,16], and other aspects. Therefore, mini-basketball has been regarded as a promising and valuable means of intervention; however, the neural mechanism by which an MBTP improves SC impairment remains unclear.

The existing evidence strongly corroborates that ASD is associated with the functional brain network, especially in the executive control network (ECN). Studies have suggested that abnormalities in the ECN may be interpreted as a neural mechanism for SC impairment in ASD. Abnormal ECN functional connectivity in children with ASD has been associated with social behavior [17]. Based on neural connectivity, evidence from a categorical–dimensional hybrid model of ASD indicated that children with ASD showed linear relations between functional connectivity of the ECN and SRS score [18]. These findings suggest that changes in functional connectivity of the ECN contribute significantly to the development of SC in children with ASD. Furthermore, the plasticity of physical exercise on the functional connectivity of the ECN has been demonstrated [19,20,21]. Therefore, exploring the effect of an MBTP on the plasticity of the ECN is the first way to reveal the neuromechanics of physical exercise to improve the social communication impairment of preschool children with ASD.

Taken together, we aimed to explore the influence of the mini-basketball intervention on SC impairment and the ECN of preschool children with ASD, so as to reveal the physical exercise intervention and improve social communication impairment and the neural mechanism of preschool children with ASD, thus providing useful and new evidence. Herein, we hypothesize that a 12-week MBTP can improve the social communication impairment and ECN of preschool children with ASD. Further, improved social communication impairment in preschool children with ASD may be associated with changes in the ECN.

## 2. Materials and Methods

### 2.1. Participants

**Figure 1:**
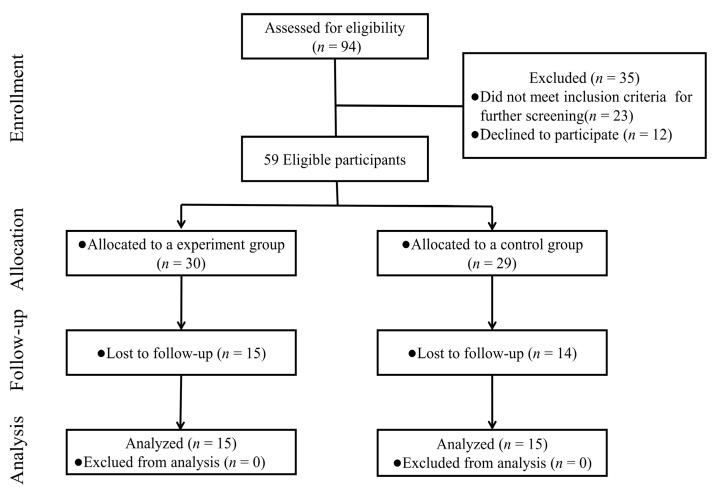
Participant follow chart.

For this study, 94 preschoolers from two educational institutions were recruited. Finally, only 30 participants completed the study (Figure 1). Inclusion criteria were as follows: (1) Han Chinese; (2) children who meet DSM-V criteria and are diagnosed with moderate to severe ASD; (3) age 3–6 years; (4) guardians of all participants agreed subjects to join the study; and (5) meeting a scan criterion for MRI. Exclusion criteria comprised of (1) head trauma; (2) children with a history of neurological disease and/or psychotic disorders; (3) hearing and vision impairments; (4) taking any medication affecting the central nervous system in the past 6 months; (5) basketball training or taking part in physical exercise regularly in the past six months; and (6) no extremity disability.

The preschool students in two rehabilitation institutions were randomized into control and experimental groups. In particular, based on the rehabilitation of routine behavior in two groups, the experimental group participated a 12-week mini-basketball intervention, while the control group only received the institutional routine behavioral rehabilitation intervention. In total, 30 subjects were entered in this research, whereby the experimental group consisted of 15 participants (12 males and 3 females), whereas the control group also included 15 (13 males and 2 females). Reasons for screening were: (1) because the parents of the children with ASD did not complete the relevant questionnaires in the post-test, 27 subjects were not considered, and (2) T1-MPRAGE was missing for 2 subjects.

Previous studies have confirmed that the degree of disease [22], sleep impairment [23], eating behavior [24], and physical health and development of children with ASD [25] affected the core symptoms of ASD. Therefore, we herein selected the Childhood Autism Rating Scale (CARS) [26], the Children’s Sleep Habits Questionnaire (CSHQ) [27], the Child Eating Behavior Questionnaire (CEBQ) [28], and the Children’s Edition of National Physical Fitness Measurement Standard Manual (General Administration of Sport of China, 2003) to evaluate the corresponding indicators, aimed to balance the above confounding variables in the experimental grouping. 

The CARS scale was filled out by the chief physician of the Child Autism Clinic in Yangzhou Maternal and Child Health Hospital following the evaluation of the clinical manifestations of children, and the evaluation doctors were consistent. The parents filled out the CSHQ and CEBQ scales according to the specific performance of subjects in daily life, and also the parents were consistent before and after filling in the scales.

### 2.2. Study Design

This study was conducted as a quasi-experimental trial that adopted a 2 × 2 mixed experimental design in which the factor “time” was within-subject factors, whereas the factor “group” was included as a between-subject factor. The protocol for this study was approved by the Ethics and Human Protection Committee of The Affiliated Hospital of Yangzhou University and registered in the Chinese Clinical Trial Registry (ChiCTR 1900024973). The parents or guardians of the study subjects signed the informed consent form before participation in the study.

### 2.3. Mini-Basketball Training Program

For this experiment, a mini-basketball training program was applied as a rehabilitation treatment to children with ASD as described previously [16,29,30,31]. The mini-basketball intervention program was mainly divided into three stages, in which each stage needed to have specific curriculum objectives; the details were as follows. (a) Stage 1 lasted for 2 weeks: to stimulate the interest of children in mini-basketball, standardize the classroom activities of children and parents (e.g., taking turns, waiting, and obeying.), and make it interesting and simple; (b) Stage 2 lasted for 8 weeks: to improve children’s mini-basketball skills (e.g., dribbling, passing, shooting, etc.) and social communication skills (includes passing, catching and relay competitions between peers); (c) Stage 3 lasted for 2 weeks: to improve the ability of children’s cooperation and collectivization, the content was based on mini-basketball group game (basketball dribbling relay, basketball passing relays, and basketball moving, shooting, etc.). 

Each class of mini-basketball lasted for 40 min, which consisted of four parts: “starting, warm-up, intervention, and relaxation.” (a) Starting: children were asked to stand in line, accept roll call and conduct class greetings, etc.; (b) warm-up: this section mainly included jogging, stretching, the movement of four limbs, etc.; (c) intervention: simple basketball training in stage 1, the mini-basketball skill learning in stage 2, the mini-basketball game in stage 3; (d) relaxation: finally, muscle relaxation and a class summary were carried out.

We set moderate exercise intensity as follows: 60–69% of maximum heart rate (MHR). The MHR was monitored using a POLAR M430 heart rate monitor and calculated with the formula: (MHR = 220 − age of the participant) [32].

### 2.4. Measurement of Social Communication

The social communication ability of preschool children with ASD was assessed using the second version of the Social Response Scale (SRS-2) [33], which is a parent or teacher rating scale developed by John N. Constantino and Christian P. Gruber in 2012 to investigate the social ability of autistic children between 2 years and 6 months to adulthood. It consisted of a total of 65 questions using a 5-point Likert scale, with higher scores indicating more severe social impairment. The SRS-2 scale was filled out by parents according to the specific performance of the subjects in daily life. Furthermore, the parents were consistent before and after filling in the scales.

### 2.5. rs-fMRI Data Collection and Processing

During the data and image acquisition process, the MRI scanner ran loudly and took a long time, so it was impossible to ensure that every subject completed the scan successfully. Therefore, to increase the success rate of the scan, this experiment moderately deprived all subjects of sleep, sedated them, and made clear specific requirements to the guardians of the children with ASD the day before the MRI scan so that the subjects could go to bed late and get up early. Each subject received an enema of 10% chloral hydrate at an interval of 6 to 8 h, 0.3 mL/kg (30 mg/kg), with a maximum dose not exceeding 10 mL. The nurse tested the level of consciousness of subjects after they fell asleep. If a mildly painful stimulus did not produce a conscious response, the subject was placed on his or her back in the MRI scanner by their guardian. Image data acquisition was performed in the MRI scanning room of the Affiliated Hospital of Yangzhou University using a 3.0T MRI scanner (GE Discovery MR750W 3.0T, Chicago, IL, USA). The data acquisition parameters for the structural MRI T1 scan were as follows: TR/TE = 7.2/3.1 ms, matrix size = 256 × 256, 170 interleaved slices, voxel size 1 mm × 1 mm × 3 mm, and FOV = 256 × 256 mm^2^ with 170 interleaved slices. The resting-state scan parameters included TR/TE = 2000/30 ms, matrix size = 64 × 64, voxel size = 3.5 mm × 3.5 mm × 4 mm, and FOV = 224 × 224 mm^2^ with 28 slices.

The ROI was selected from the executive control brain network template (https://findlab.stanford.edu/functional_ROIs.html (accessed on: 1 October 2020)) of the center for functional imaging of neuropsychiatric diseases (Table 1).

### 2.6. Procedure

The whole experiment consisted of three parts: pre-test, mini-basketball intervention, and post-test (Figure 1).

Pre-test: This part was performed one week before the formal experiment. We communicated entirely with the parents of the subjects and introduced the mini-basketball intervention to all children with ASD and their parents. At the same time, the demographic information of the subjects was investigated in detail, and their parents and evaluation doctors filled out the relevant questionnaires.

Mini-basketball intervention: In the experimental group, preschooler children with ASD were given a moderate-intensity mini-basketball training program for 40 min a day, 5 days a week, for 12 weeks, while the control group received rehabilitation of routine behavior.

Post-test: After completing the 12-week mini-basketball intervention, we then collected brain image data from preschool children with ASD. Finally, parents filled out the SRS-2 scale, and the parents who filled out the scale were the same person as the pre-test.

### 2.7. Statistical Analysis

First, a homogeneity test was performed on the demography data (age, sex, and BMI), as well as the CARS, physical assessment, CSHQ scores, and CEBQ scores of the experimental and control groups using an independent sample t-test with SPSS statistical software, version 22.0 (IBM, Armonk, NY, USA). The results are expressed as mean ± standard deviation (M ± SD). Second, repeated measures analysis of variance (ANOVA) was used to analyze the social communication impairment data, whereas effect sizes are presented as partial eta-squared (partial η^2^). When a significant interaction was found, a simple effects analysis was conducted.

We employed the DPABI (http://rfmri.org/dpabi (accessed on: 1 October 2020)) software to calculate the functional connectivity of ECN, while a two-sample t-test based on GRETNA (http://www.nitrc.org/projects/gretna/ (accessed on: 1 October 2020)) was used to test whether the functional connectivity between the 12 pairs of ROIs examined in the two groups of preschool children with ASD were homogeneous in pre-test. We also performed repeated measurement ANOVA on the functional connectivity between the 12 ROIs measured before and after in the two groups of preschool children with ASD using MATLAB software (MathWorks, Natick, MA, USA). Lastly, the brain regions with significant differences were obtained, and subsequently, their functional connectivity values were extracted to explore the changes in their intensity.

Finally, correlation analysis was used to examine the difference between the FC value of the ECN and the difference between the social communication performance, before and after 12-week MBTP.

## 3. Results

### 3.1. Participant Characteristics

The demographic characteristics of the participants are summarized in Table 1. We found that participants exhibited no difference at baseline in terms of gender (Chi-square: χ^2^ = 0.24, *p* = 0.62), age (t (28) = 1.60, *p* = 0.12 > 0.05), BMI (t (28) = −0.25, *p* = 0.81 > 0.05), speed-agility (t (28) = 1.22, *p* = 0.23 > 0.05), muscular strength (t (28) = 1.45, *p* = 0.16 > 0.05), balance ability (t (28) = 0.21, *p* = 0.84 > 0.05), CARS (t (28) = −0.94, *p* = 0.36 > 0.05), CSHQ (t (28) = −0.62, *p* = 0.54 > 0.05), and CEBQ (t (28) = 0.14, *p* = 0.89 > 0.05) during the study period. Mean and standard deviation of physical fitness performance and the SRS-2 scores of all children are depicted in Table 2.

### 3.2. Social Communication Performance

Here, we adopted the repetitive measure analysis of variance method to explore the influence of the MBTP on the SRS-2 score of preschool children with ASD. The results of the interaction between groups and times can reveal whether the MBTP causes a change in the SRS-2 score. We found a significant group × time interaction on social communication total score (F (1, 28) = 7.77, *p* = 0.009 < 0.01, partial η^2^ = 0.22), social cognition (F (1, 28) = 9.97, *p* = 0.004 < 0.01, partial η^2^ = 0.26), and social communication (F (1, 28) = 8.40, *p* = 0.007 < 0.01, partial η^2^ = 0.23). Analysis of simple effects according to values (Table 2) revealed that the pre-test scores in all SRS-2 dimensions of the experimental and control groups were homogeneous (*p* > 0.05). However, there were significant differences in the total score of SRS-2 (*p* = 0.03 < 0.05) and the score of social communication dimension (*p* = 0.02 < 0.05) between the pre-test and post-test in the control group. In comparison, the score of the post-test was higher than that of the pre-test in the control group. Similarly, there was a significant difference between the pre-test and post-test scores of the experimental group in the dimension of social cognition (*p* = 0.01 < 0.05). Comparatively, the post-test score was lower compared with that of the pre-test score in the experimental group (A higher score means worse symptoms) (Table 3).

### 3.3. Executive Control Network

There was no significant difference in 12-pair ROI–ROI for the ECN in preschool children with ASD at baseline. We used ANOVA statistical function in MATLAB to perform repeated measures analysis of variance of the functional connectivity matrix for the 12 regions of interest (ROI) in the ECN generated by each participant. We subsequently observed significant differences in functional connectivity of 2-pair ROI–ROI in the ECN: left inferior frontal gyrus and right cerebellum (*p* = 0.03 < 0.05), left middle temporal gyrus and right cerebellum (*p* = 0.01 < 0.05) (Table 4).

**Figure 2:**
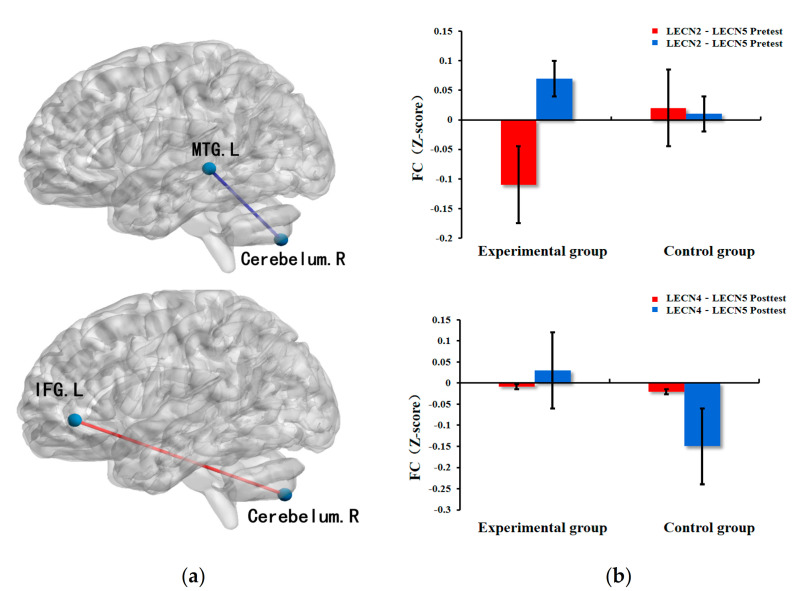
ECN changes caused by exercise intervention. Note: (**a**) left middle frontal gyrus, MFG.L, left middle temporal gyrus, MTG.L, right cerebellum, R, the blue nodes indicate the left ECN, the red line indicates FC enhancement, the blue line indicates FC decreased; (**b**) difference in FC values of ECN between the experimental group and the control group.

The mean comparison of the functional connectivity between the two pairs of ROIs demonstrated that the functional connectivity between the left inferior frontal gyrus and right cerebellum of the ASD children in the experimental group were enhanced, while the functional connectivity between the left middle temporal gyrus and right cerebellum were decreased in the control group (Figure 2).

### 3.4. Correlation between Functional Connectivity of ECN and Behavioral Performance

Correlation analysis was used to examine the difference between the FC value of ECN and the difference between the social communication performance, before and after 12-week MBTP. In order to avoid confusing the influence of variables, we took the height, weight, BMI, CARS score, CEBQ score, and CSHQ score as control variables for analysis. Our results showed the difference between the FC of the right cerebellum and left inferior frontal gyrus was not significantly correlated with the difference in the social cognition (r = −0.20, *p* = 0.35), social communication (r = −0.14, *p* = 0.52), total scores of SRS-2 (r = −0.16, *p* = 0.46), the difference between the FC of right cerebellum and left middle temporal gyrus was not significantly correlated with the difference in the social cognition (r = −0.29, *p* = 0.16), social communication (r = −0.32, *p* = 0.13), total scores of SRS-2 (r = −0.25, *p* = 0.24). 

## 4. Discussion

This study was designed to explore the effects of 12-week MBTP on SC and functional connectivity of the ECN in children with ASD. In particular, one group, the experimental group, received a 12-week MBTP based on routine rehabilitation, while the other group, the control, received only conventional rehabilitation. In this work, we also tried to control the confounding variables as much as possible. Therefore, the findings of this study can reveal the neural basis of SC improvement due to the mini-basketball training program.

### 4.1. Behavioral Performance

In the present investigation, we found that 12-week MBTP improved the social communication impairment of preschool children with ASD. Previous studies have also elucidated that exercises such as mini-basketball [15], aquatic program [34], and Judo program [35] intervention can improve the social communication impairment of children with ASD. Thus, physical exercise is a promising method of rehabilitation for children with autism, and is worthy of further research. 

Notably, the MBTP significantly improved the social cognition and social communication sub-dimensions of children with ASD. There are several plausible explanations for these observations. For example, research has shown that social cognition is the ability to recognize, manipulate, and respond to socially relevant information, thus creating complex performance characteristics [36]. Therefore, the improvement of the social cognitive ability of preschoolers with ASD may perhaps be due to the process of the MBTP because children need to identify the demonstration actions of teachers to imitate, which is also a process of social information processing [37]. Besides, a large number of social factors was contained in the process of the MBTP, such as children needing to accept roll call, conduct class greetings, pass and catch between peers, etc.; participation in this could provide opportunities to establish social relationships with their coach or peers, to enhance their social skills [38]. Recent studies tend to support this view, and hence children need to watch and cooperate with their peers in order to increase their social behavior such as in karate training [10]. Similar findings have been reported in water exercise swimming programs, containing a rich social communication environment [8]. 

We also identified no significant improvement in social motivation, autistic mannerisms, and social awareness. However, in one study, horseback riding intervention was found to markedly improve social motivation in children with ASD [11]. Thus, horseback riding was considered a rewarding stimulus, and children with ASD needed stronger motivation to participate, while the MBTP mainly focused on fine movement. This result may be ascribed to differences in the content of the intervention program. Additionally, autistic mannerisms and social awareness showed a trend of improvement although it was not statistically significant. We therefore assume that extending the period of the MBTP may cause a more significant effect, but more evidence is needed to verify this conjecture.

Interestingly, we have obtained some meaningful speculations. The content of the MBTP contained multiple elements of intervention; children with ASD were not only required to run, jump and other basic movements, but also to touch the ball and respond to instructions and other multifaceted stimulation exercises during this procedure; therefore, an MBTP may be beneficial to the development of the sensory and perceptual ability of children with ASD. The children need learn new movement and master the connection between movements in the process of the MBTP, which required a high level of cognition and control, and this may be beneficial for improving cognition function in children with ASD. In short, an MBTP may play a positive role in improving ASD-like behaviors. Collectively, based on the above findings, we uncovered that the MBTP played a positive role in improving social communication impairment in children with ASD, and considering factors such as program design and the content of the intervention cycle may also influence the intervention. Therefore, in future studies, there is a need to establish a more perfect intervention program to evaluate the efficacy of the improvement.

### 4.2. Executive Control Network

Evidence from exercise and brain plasticity shows that physical exercise can cause plasticity changes in the functional connectivity of the ECN [21,39]. Remarkably, our results support existing evidence that exercise intervention induces changes in the functional connectivity of the ECN. We found an increase in functional connectivity of the ECN between the right cerebellum and left inferior frontal gyrus. We subsequently inferred that this result might be attributed to the MBTP. 

Multiple recent studies have shown that brain development in children with ASD has poor connectivity between regions; among them, there is evidence that the frontal and subcortical cerebellar regions exhibit inadequate connectivity [40,41]. Herein, we found enhanced functional connectivity in the right cerebellum and left inferior frontal gyrus in preschoolers with ASD after 12-week MBTP, suggesting that exercise intervention optimized the abnormalities of functional connectivity in ECN. Several studies have demonstrated that the cerebellum is a key brain region involved in motor function such as coordination and balance [42,43]. Further, the cerebellum is functionally connected to the frontal lobe [44], and evidence of co-activation of the cerebellum and the frontal lobe has also been found in functional neuroimaging [45,46]. Therefore, the enhanced cerebellar and frontal functional connectivity in preschoolers with ASD is the result of the 12-week MBTP. 

In addition, studies have identified that children with ASD exhibit developmental disorders in the temporal lobe [47], and the functional connectivity between the temporal lobe and other brain regions decreases with age compared with normal children [48]. In this study, compared with the control group, the MBTP enhanced the functional connectivity between the left middle temporal gyrus and right cerebellum in the experimental group of preschoolers with ASD, thereby inhibiting the injury trend of functional connectivity deficiency. Similarly, previous studies have also confirmed that exercise can improve the abnormal functional connectivity between the temporal lobe brain region and related brain regions [19,49], which provides supportive evidence for the results of this study. Overall, this study found a positive effect of exercise on ECN in preschoolers with ASD, providing valuable and novel evidence for exercise and plasticity of the brain on children with ASD.

### 4.3. Neural Mechanism of MBTP on Social Communication Impairment in Preschool Children with ASD

Our finding revealed that there was no statistically significant correlation between the improvement of functional connectivity within the ECN and the improvement of SC, and it was inconsistent with previous studies. Several reasonable explanations that might answer this question exist. For instance, ECN-related brain regions, such as the cerebellum, frontal lobe and temporal lobe, are all involved in the formation of the “social brain” network [50,51,52], and many studies have also suggested that these brain regions are the neural basis of social communication impairment in ASD [18,53,54,55,56,57,58]. Elsewhere, functional connectivity in the right prefrontal, posterior, and inferior frontal gyrus was negatively correlated with social communication impairment scores. Moreover, longitudinal studies indicated that mini-basketball can promote social development by improving the structure of white matter [9]. Regrettably, the current study hoped to explore the neural basis of improving social communication impairment by mini-basketball from the perspective of functional brain network, but there was no statistically significant correlation between improved functional connectivity of the ECN and better performance of social communication impairment in our results. Fortunately, both showed a positive trend of improvement, which indicated that the neural mechanism of the MBTP on social interaction disorder of autistic preschool children may be the improvement of the ECN. We surmised that the reason for it not being statistically significant lies in small sample size or an imperfect MBTP. Thus, future studies will be further explored and refined, and reveal the functional brain network mechanism of the MBTP to improve social communication.

### 4.4. Limition

We believe that the limitations of this study is worth further discussion. First, although MBTP has a positive impact on the SC impairment of preschool children with ASD, it does not seem to be comprehensive (no significant statistically improvement in social motivation, social awareness sub-dimensions were shown). In the future research, we still need to think deeply and optimize our own program, hoping to play a more effective role in the rehabilitation of children with ASD. Second, it is a subjective measure that SRS-2 was administered by parents of the participating children, but we must admit that there are no other tools to help us evaluate ASD children more objectively.

## 5. Conclusions

In summary, this study demonstrates that social communication impairment was improved, and functional connectivity of the ECN was optimized after establishing an exercise regimen in preschool children with ASD. Notably, an MBTP is a valuable exercise intervention to improve social communication impairment, especially in children with ASD; this work provides an important baseline for future research in exploring the neural mechanisms by which exercise improves social communication impairment. Finally, our study has important implications for practice to the development and application of rehabilitation programs for children with ASD. 

## Figures and Tables

**Table 1:** Twelve ROIs of executive control network.

ROI	Brain Hemispheres	Primary Regions	Brodmann’ Area
RECN1	Right	orbitofrontal gyrus/dorsolateral prefrontal cortex	8/9
RECN2	Right	frontal pole/inferior frontal gyrus	10/46/47
RECN3	Right	inferior parietal lobule	7/39/40
RECN4	Right	middle temporal gyrus	20/37
RECN5	Left	cerebellum	–
RECN6	Right	caudate nucleus	–
LECN1	Left	orbitofrontal gyrus/dorsolateral prefrontal cortex	8/9/46
LECN2	Left	frontal pole/inferior frontal gyrus	10/45/47
LECN3	Left	inferior parietal lobule	7/39/40
LECN4	Left	middle temporal gyrus	20/37
LECN5	Right	cerebellum	–
LECN6	Left	thalamus	–

**Table 2:** Participant demographics evaluation results (M ± SD).

	Control Group	Experiment Group
*N*	15	15
Gender(male/female)	12/3	13/2
Age	5.03 ± 0.55	4.67 ± 0.70
BMI	15.75 ± 0.67	15.88 ± 1.80
CARS ^a^	38.13 ± 4.47	39.80 ± 5.24
CSHQ ^b^	56.47 ± 5.04	58.60 ± 12.29
CEBQ ^c^	55.20 ± 8.25	54.40 ± 20.05
20-m shuttle run (s)	13.31 ± 4.16	11.83 ± 2.23
Standing long jump (cm)	50.23 ± 28.75	37.00 ± 21.86
Sit-and-reach (cm)	17.55 ± 6.09	19.61 ± 2.43
Balance beam (s)	21.92 ± 36.00	19.90 ± 11.10

Note: ^a^ CARS: Childhood Autism Rating Scale; ^b^ CSHQ: Children’s Sleep Habits Questionnaire; ^c^ CEBQ: Child Eating Behavior Questionnaire.

**Table 3:** Analysis of two groups for social communication variables (M ± SD).

	Experiment Group (*n* = 15)		Control Group (*n* = 15)	
	Baseline	Post-Test	Baseline	Post-Test
SRS-2 T-score	89.70 ± 25.89	81.50 ± 4.55	85.30 ± 20.04	97.30 ± 21.35
Social cognition	19.50 ± 5.59	16.50 ± 6.07	17.30 ± 3.56	19.30 ± 3.89
Social communication	31.70 ± 1.86	28.40 ± 9.66	30.90 ± 8.06	35.70 ± 8.09
Social motivation	14.40 ± 4.72	12.30 ± 3.84	14.40 ± 4.39	14.50 ± 4.19
Autistic mannerisms	12.30 ± 5.04	13.20 ± 5.62	12.30 ± 5.36	16.40 ± 6.15
Social awareness	11.90 ± 2.80	11.20 ± 3.61	10.50 ± 2.45	11.40 ± 2.59

**Table 4:** Function connectivity results for 12 ROI–ROI changes in ECN, segregated by time point.

	RECN1	RECN2	RECN3	RECN4	RECN5	RECN6	LECN1	LECN2	LECN3	LECN4	LECN5	LECN6
RECN1												
RECN2	0.67											
RECN3	0.51	0.19										
RECN4	0.73	0.96	0.48									
RECN5	0.77	0.71	0.80	0.37								
RECN6	0.76	0.22	1.00	0.61	0.90							
LECN1	0.45	0.90	0.63	0.24	0.61	0.87						
LECN2	0.78	0.34	0.75	0.76	0.40	0.73	0.41					
LECN3	0.38	0.77	0.57	0.65	0.46	0.45	0.41	0.87				
LECN4	0.48	0.91	0.08	0.81	0.67	0.66	0.24	0.25	0.20			
LECN5	0.17	0.06	0.30	0.16	0.78	0.76	0.50	0.03 *	0.05	0.01 *		
LECN6	0.05	0.79	0.73	0.61	0.32	0.81	0.73	0.43	0.31	0.98	0.50	

Note: “*” indicates *p* < 0.05 (uncorrected).

## Data Availability

The data presented in this study are available on request from the corresponding author.
